# The trajectory of symptom burden in exposed and unexposed survivors of a major avalanche disaster: a 30 year long-term follow-up study

**DOI:** 10.1186/s12888-019-2159-7

**Published:** 2019-06-10

**Authors:** Lars-Petter Bakker, Milada Cvancarova Småstuen, Jon Gerhard Reichelt, Christer Lunde Gjerstad, Arnfinn Tønnessen, Lars Weisæth, Pål Herlof Herlofsen, Ellen Karine Grov

**Affiliations:** 1 Norwegian Armed Forces Joint Medical Services, Institute of Military Psychiatry, PO Box 1550, Sentrum, N-0015 Oslo, Norway; 2 Department of Nursing and Health Promotion, Faculty of Health Sciences, Oslo Metropolitan University, PO Box 4, St. Olavs plass, N-0130 Oslo, Norway; 3 Institute of Clinical Medicine, Division of Mental Health and Addiction, University of Oslo, Gaustad sykehus. Bygg 4, PO Box 1039, N-0315 Oslo, Blindern Norway; 4 Tranby, Norway

**Keywords:** Disaster, Avalanche, Posttraumatic stress symptoms, Anxiety, Mental health symptoms, Long-term follow-up

## Abstract

**Background:**

Limited research exists concerning the long-term effects of avalanches on survivors’ mental health beyond the first years after the accident. The aims of this study were to describe and evaluate possible differences in long-term mental health symptoms after a major avalanche disaster between exposed and unexposed soldiers using a longitudinal design.

**Method:**

Present mental health symptoms were examined among avalanche exposed (*n* = 12) and unexposed (*n* = 9) soldiers by PTSS-10, IES-15 and STAI-12 in four waves (1986–1987 and 2016).

**Results:**

Binary logistic regression revealed that the odds to score above the cut-off were significantly lower for both groups after one year compared to baseline for PTSS-10 (*p* = 0.018) and significantly lower after 30 days compared to baseline for IES-15 (*p* = 0.005). Data did not reveal significant differences between the exposed and unexposed groups regarding adjusted PTSS-10, IES-15 or STAI-12 mean scores compared. Linear mixed model-analyses revealed significant effects of time. The adjusted mean scores declined over time for both groups: PTSS-10 (*p* = 0.001), IES-15 (*p* = 0.026) and STAI-12 (*p* = 0.001), and the time trajectories for PTSS-10 were significantly different between the groups (*p* = 0.013). Although not significant (all *p* > 0.05), results indicated that a larger proportion of soldiers in the exposed group experienced posttraumatic stress symptoms (5/12) (PTSS-10 score ≥ 4) and distress symptoms (6/12) (IES-15 score ≥ 26) above cut-off points, 30 years post-disaster.

**Conclusions:**

The course of mental health symptoms may persist, and even increase, in selected and trained military personnel 30 years after exposure to a natural disaster. These findings may be of great importance for health authorities planning appropriate follow-up.

## Background

Posttraumatic stress (PTS) may persist long after exposure has ended [1]. It is well documented that soldiers fighting in World War II, Afghanistan, and concentration camp survivors, might suffer from negative long-term health effects after trauma [2–5]. The risk of developing posttraumatic stress disorder (PTSD) is related to exposure to potentially traumatic events (PTEs). However, the incidence and prevalence vary with the type and duration of exposure; exposure to premeditated traumas is associated with the highest prevalence rate: interpersonal events such as physical threat (weapon), childhood abuse, rape, imprisonment, sexual abuse, kidnapping or being taken hostage and verbal threat/violence from close relations [6–11]. Exposure to PTEs is described as common in most epidemiological surveys of PTSD in numerous countries. Studies have shown that between 20 and 90% of the general population will once in their life experience a PTE [6, 12, 13], and estimates of lifetime prevalence rates of PTSD are between 1.3 to 11.2% [6, 7, 13, 14].

A recent study on the epidemiology of PTSD in Norway aimed to assess lifetime incidence and prevalence of exposure to PTEs and PTSD in a sample representative of the Norwegian population [7]. Lassemo and colleagues [7] claim that lifetime prevalence of Norwegian men at risk of being exposed to a natural catastrophe exemplified as a form of PTE is 1.4%, and of those, 9.1% will probably fullfill the diagnostic criteria for being at risk for PTSD.

Studies on the long-term effects of disasters are limited, but the majority indicate that survivors may experience a range of negative health effects. PTSD is one of several psychiatric conditions that can be observed after trauma [15–17]. However, a broad range of other mental health conditions may develop in the wake of trauma, such as depression [17, 18], sleep-related disturbances and chronic anxiety [1, 17, 19–21], and suicidal behavior [22, 23], but trauma exposure has also been associated with reduced quality of life, impaired psychosocial functioning [24], and increased physical health problems [20, 25–30]. Finally, alcohol abuse is often associated with poor physical health and PTSD [31–33].

Neria, Nandi, and Galea [34] and Galea, Nandi, and Vlahov [35] argue that PTSD is one of the most common post-disaster mental health problems. According to Galea and colleagues [35], 5 to 60% of exposed survivors will be affected by PTSD. However, some researchers claim it is better to compare the effects of disasters of the same nature, rather than group different disasters into the same category, as reactions to disasters may be influenced by incident type, location, causes and context [36, 37].

Natural disasters like avalanches allow examination of exposure to a well-defined stressor. Avalanche accidents leave a survivor sample which has been directly exposed to overwhelming death threats. However, not many long-term avalanche studies have been conducted, and findings are limited to the first year post-disaster [20].

To our best knowledge, only four studies exist in the literature on short-term mental health effects of being exposed to avalanche disasters: two in Iceland and two in Norway.

The Icelandic studies examine two different avalanche-exposed communities in small fishing villages the first year post-disaster. These studies indicated that approximately 40% of survivors suffered from PTSD 10 weeks to 14 months after the avalanches [38, 39].

Two Norwegian studies have assessed PTSD prevalence in soldiers who survived an avalanche, during the first year post-disaster [40, 41]. Herlofsen’s [40] and Johnsen and colleagues [41] indicate that a substantial proportion of survivors suffered from PTSD-symptoms up to four months post-disaster. Herlofsen’s [40] presents data from the first three waves of the present study. Our study aims to compare data presented by Herlofsen’s [40] with assessment 30 years post-disaster.

To our best knowledge, only one study exists on long-term health effects after avalanches. This study was conducted in Iceland to follow up the studies done by Asmundsson and Oddsson [38] and Finnsdottir and Elklit [39], and has a 16-year follow-up of the survivors [20, 21, 42, 43].

Thordardottir and colleagues [20] and Thordardottir, Hansdottir, Valdimarsdottir, and colleagues [21] reported avalanche-specific PTSD-symptoms in 16% of survivors (respectively 12% in men and 19% in women).

In the current study we have examined the 30 year trajectory of mental health symptoms after exposure to an avalanche. This presentation is unique, particulary regarding the follow-up time. We studied mental health symptoms, i.e., PTS, distress and anxiety symptoms, and compared the exposed and unexposed Vassdalen soldiers 30 years post-disaster.

We anticipated that the pattern of change for all outcome variables would develop differently across time depending on whether the responders were in the exposed or unexposed group.

## Method

### Participants

During the two weeks preceding March 5, 1986, the weather conditions in Vassdalen, in Northern Norway, had deteriorated. The changes in weather conditions resulted in increased avalanche risk in the area where the NATO exercise Anchor Express 1986 was scheduled. A few minutes past 1:00 p.m. an avalanche struck a platoon of 31 soldiers from an engineering corps, leaving 16 dead and 15 survivors [40].

All survivors (exposed) (*n* = 15), and the remaining members (unexposed) (*n* = 15) of the platoon who were stand-by outside the avalanche area, were enrolled in the study immediately following the disaster. The unexposed soldiers were included in the study as a comparison group.

When the follow-up study was conducted, 30 years later (2016–2017), the platoon’s exposed or unexposed soldiers were all alive and traceable (*N* = 30). The response rate was 80% for the exposed group (12/15) and 60% (9/15) for the unexposed group.

### Study design and procedure

This unselected, longitudinal study was designed to compare changes in mental health symptoms (i.e., PTS, distress and anxiety symptoms) among exposed and unexposed soldiers over time. Data were collected at four measuring points, Time1-Time4 (T1-T4), over 30 years. The three first measuring points (T1-T3) aimed to assess mental health symptoms, and data were collected within the first 375 days post-disaster; T1 after 4 days, T2 after 30 days and T3 at 375 days post-disaster. The fourth measurement (T4) was conducted 30 years post-disaster.

By law, the Norwegian Armed Forced Joint Medical Services’ record has an overview of the sample in this survey. Information about the survey, and the questionnaire, with a sheet to sign for written consent was sent by postal mail to all potential participants. They were informed that answering and returning the questionnaire and the signed consent form, were considered as a consent to participate in the study. The participants were followed up by a phone call and a message via mail or postal mail thanking those who had returned the questionnaire and reminding those who had not returned the questionnaire to consider doing so. Participants needing professional psychiatric aid were offered support from the Institute of Military Psychiatry. All participants were told that they could withdraw whenever they wanted during the survey, without any further explanation and that withdrawal would not affect their contact with the Norwegian Armed Forced Joint Medical Services in the future.

### Measures

#### Background information

For this particular study, PTEs were assessed in addition to demographic and background information at the last wave (T4). For details, see Table 1.

**Table 1:** Characteristics of soldiers exposed and unexposed to the avalanche at Vassdalen in 1986

	Exposed (*n* = 12)	Unexposed (*n* = 9)	*P*-values
Age			0.980^1^
Mean age (SD)	52.4 (0.87)	52.4 (0.91)	
Mean age at time of avalanche (SD)	20.5 (0.87)	20.5 (0.91)	
Median age	52.3	52.3	
Median age at time of avalanche	20.5	20.5	
	n (%)	n (%)	
Education			0.135^2^
University	5 (42)	4 (44)	
High school or trade school	5 (42)	5 (56)	
Grade school	2 (16)	0 (0)	
Current living situation			0.154^2^
Married or in a relationship	7 (58)	7 (78)	
Single, divorced or widowed	5 (42)	2 (22)	
Employment status			0.603^3^
Working	9 (75)	8 (89)	
On disability	3 (25)	1 (11)	
Children			0.378^2^
0	2 (17)	1 (12)	
1–2	8 (66)	4 (44)	
3–4	2 (17)	4 (44)	
Did the disaster affect your physical health negatively?			0.005^3^
Yes	8 (67)	0 (0)	
No	4 (33)	9 (100)	
Did the disaster affect your mental health negatively?			0.024^3^
Yes	8 (67)	1 (11)	
No	4 (33)	8 (89)	
Any suicidal thoughts since the accident?			1.000^3^
Yes	2 (13)	1 (11)	
No	10 (67)	8 (89)	
Any PTEs before or after the accident?			0.673^3^
Yes	8 (67)	5 (56)	
No	4 (33)	4 (44)	

^1^T-test (2-tailed, equal variances assumed)

^2^Pearson chi-square (2-sided)

^3^Fisher’s exact test (2-sided)

#### Posttraumatic symptom Scale-10 (PTSS-10; Holen, Sund [44])

The PTSS-10 comprises a 10-item self-report questionnaire, originally developed by the Division of Disaster Psychiatry (at the Armed Forces Joint Medical Service in Oslo, Norway) [44]. The scale covers general stress manifestations such as irritability, sleep difficulties, depressed mood and startle reactions. PTSS-10 response alternatives is usually given on a seven point Lickert scale from 1 rarely/seldom to 7 often. In the current study the response alternatives were dichotomous; not present - No(0), and present - Yes(1). The PTSS-10 sum scores constitute the summation of the ratings (score range = 0–10), the total sum being interpreted according to the two following levels of PTS-symptoms: 0 to 3 (mild/moderate range) and 4 to 10 (moderate/severe range). Most often a score of 6 or more represent “case” and 4–5 represent “caseness”. In the current study a cut-off point of 4 or above indicates a need for psychological referral.

This measure has demonstrated satisfying validity, reliability and internal consistency [44–47]. The PTSS-10 provides good face validity, and the direct wording of the items was closely related to the PTSD diagnostic criteria. The PTSS-10 was used at all four waves (T1-T4). Participants were asked to report current PTS-symptoms.

#### Impact of event Scale-15 (IES-15; Horowitz, Wilner [48])

The IES-15 is a self-report measure designed to assess current subjective distress and PTS-symptoms for any specific life event [48, 49]. The scoring method for measuring distress used a 6-point scale: 0; not at all, 1; rarely, 2; somewhat, 3; sometimes, 4; very much so, and 5; often. The 15-items scale provides a total distress score and two sub-scores: Intrusion (7 items) (range = 0–35) and Avoidance (8 items) (range = 0–40). Scores from 0 to 8 indicate low level of distress, 9–19 represent moderate distress and 20 or more, high level of distress, in both subscores. High levels of distress indicate need of professional evaluation and possible treatment while moderate levels of distress are considered cause for concern [50]. The total distress score (score range = 0–75) represents the summation of the constructions Intrusion and Avoidance. The instrument is closely connected with symptoms of PTSD [51]. The present study used IES-15 to detect distress and PTS-symptoms in all four data collection waves. The total distress score can be interpreted according to the following four levels of PTS-symptoms: 0 to 8 (subclinical range), 9 to 25 (mild range), 26 to 43 (moderate range), 44 and higher (severe range) [51]. Sterling [51] suggests that cut-off points of 26 or above indicate psychological referral.

The IES-15 has demonstrated acceptable validity, reliability and internal consistency [48, 49], but does not include the third major cluster of PTSD-symptoms, a hyperarousal subscale [51].

Participants were asked to report current intrusion and avoidance symptoms during the past two weeks.

#### State anxiety/aggression Inventory-12/18 (STAI-12/18; Spielberg, Gorsuch [52])

The STAI-18 is a self-report questionnaire designed to measure the presence and severity of current symptoms of anxiety and generalized propensity to be anxious and aggressive. The version used at all four data collection waves (T1-T4) contained only the 12 anxiety items. Data for the dimension aggression were not used due to missing data (6 items). In the present study STAI-18 will be named STAI-12.

The values measuring anxiety relate to a 4-point scale; 1; not at all, 2; somewhat, 3; moderately so, and 4; very much so. The STAI-12 sum scores represent the summation of the ratings (score range = 12–48), and cut-off points of 30 or above would be grounds for psychological referral.

The instrument STAI-18 has demonstrated satisfying validity, reliability and internal consistency [52–54]. Participants were asked to report current symptoms of anxiety.

### Statistical analyses

The sample is decribed using descriptive statistics. Continuous variables are decribed with mean (*M*) and standard deviation (*SD*), categorical ones with counts and percentages. Possible crude differences between groups (exposed and unexposed) at T1-T4 were assessed using the Wilcoxon-Mann-Whitney test for continuous variables and Chi-square or Fisher’s exact test for categorical variables.

Further, for the continuous variables, linear mixed model (LMM) regression analyses were used to estimate possible differences between groups over time. An unstructured covariance matrix was specified to accommodate for heterogeneous residual variances across time. Restricted maximum likelihood estimation was used to produce unbiased estimates of the model parameters. All overall effects were analysed using *F* tests. The results were presented as estimated *Ms* with 95% confidence intervals (CI). Least significant difference post hoc tests were used to compare *Ms* at given time points. All models were fitted with group, time and group*time interaction terms. The model fit for regression models was good and the residuals followed normal distribution.

All outcome measures were dichotomized and odds for scoring over a given cut-off value were modeled using binary logistic regression models for repeated measures. The models were fitted with group and time. The results were expressed as odds ratios (OR) with 95% CI. All tests were two-sided and *p*-values < 0.05 were considered statistically significant. We regarded our study as an exploratory analysis and did not adjust for multiple testing.

Data were analysed using the statistical program IBM SPSS Statistics version 24.0 [55] and Stata version 14.2 (StataCorp, 2005).

## Results

The exposed and unexposed soldiers reported almost similar numbers of experienced PTEs in their lifetime (*p* > 0.05) (Table 1). In the exposed group 8/12 (67%) reported one or more PTE. For the unexposed group, 5/9 (56%) reported one or more PTE (Table 1).

Most of the remaining background characteristics were similar in both groups except exposed group self-affection for the disaster’s negative impact on physical (*p* = 0.005) and mental health (*p* = 0.024) (Table 1).

Inspection of unadjusted *M*-values for PTSS-10, IES-15 and STAI-12 scores indicated different patterns between the two groups, especially for PTSS-10 and IES-15, from T1 to T4. However, these changes did not reach the level of statistical significance using Wilcoxon-Mann-Whitney test (all *p* > 0.05*,* data not shown) (all unadjusted *Ms,* see Table 2).

**Table 2:** Measures of mental health problems over time in soldiers exposed and unexposed to the avalanche at Vassdalen in 1986

Measure	Exposed					Unexposed				
	*n*	*M*	*SD*	*Md*	Caseness (%)	*n*	*M*	*SD*	*Md*	Caseness (%)
PTSS-10										
T1 (4 days)	15	2.80	2.5	3.00	5 (33)	15	4.20	2.4	4.00	10 (67)
T2 (30 days)	12	2.42	2.5	2.00	2 (17)	13	3.15	2.5	3.00	5 (38)
T3 (375 days)	15	1.80	1.7	1.00	5 (33)	15	0.93	1.5	0	1 (7)
T4 (30 years)	12	3.75	3.4	2.50	5 (42)	9	1.33	2.4	0	1 (11)
IES-15										
T1 (4 days)	15	18.47	12.3	14.00	6 (40)	14	24.80	12.5	26.00	8 (53)
T2 (30 days)	12	14.75	15.9	9.50	2 (17)	13	13.54	6.0	14.00	0 (0)
T3 (375 days)	15	18.53	13.1	15.00	4 (27)	15	15.40	10.1	15.00	3 (20)
T4 (30 years)	12	25.92	23.9	22.50	6 (50)	9	9.67	12.5	4.00	1 (11)
STAI-12										
T1 (4 days)	15	20.73	7.5	18.00	0 (0)	15	25.07	7.1	24.00	1 (7)
T2 (30 days)	12	20.00	9.1	16.00	1 (8)	13	19.77	5.9	19.00	2 (15)
T3 (375 days)	15	17.47	4.2	18.00	0 (0)	15	15.87	5.0	14.00	0 (0)
T4 (30 years)	12	18.67	4.3	19.50	0 (0)	9	16.67	6.5	14.00	1 (11)

Note. *M, SD*, and *Md* are all unadjusted

The exposed group indicated a decrease in almost all unadjusted *M*-values from T1 to T3; however, the *M*-scores for PTSS-10 and IES-15 increased again 30 years post-disaster (T4), (Table 2). The PTSS-10 and IES-15 *M*-scores for the exposed at T4 were above any earlier measured unadjusted *M*-scores (T1-T3). The anxiety *M*-scores (STAI-12) indicated a decrease from T1-T3; however, the *M*-value at T4 increased again, but not above the previous (T1-T3) measured values (Table 2).

For the unexposed group, our data revealed a decrease in almost all unadjusted *M*-values from T1 to T3, with T4 indicating a very small increase in *M*-scores for PTSS-10 and STAI-12. For all waves, the IES-15’s lowest *M*-score was measured at T4 for the unexposed .

The unexposed group seems to be doing better than the exposed group both for the first year post-disaster (T1-T3) and 30 years post-disaster (T4) regarding unadjusted *M*-levels of mental health symptoms. All (T1-T4) reported unadjusted *M*-scores and *SDs* are listed in Table 2.

LMM analyses did not reveal any statistically significant differences between the groups in adjusted *Ms* for mental health scores when assessed with PTSS-10, IES-15 and STAI-12 when all measurements were considered (adjusted *Ms/SD/*95%CI see Table 3).

**Table 3:** Linear mixed model

	Exposed			Unexposed		
	*M*	*SE*	95% CI	*M*	*SE*	95% CI
PTSS-10						
T1	2.80	0.63	1.50–4.10	4.20	0.63	2.90–5.50
T2	2.07	0.67	0.69–3.45	3.19	0.66	1.83–4.55
T3	1.80	0.42	0.94–2.66	0.93	0.42	0.08–1.79
T4	3.44	0.86	1.64–5.24	2.13	0.94	0.19–4.06
IES-15						
T1	18.47	3.20	11.91–25.03	24.80	3.20	18.24–31.36
T2	14.40	3.27	7.68–21.12	13.41	3.19	6.83–19.99
T3	18.53	3.02	12.35–24.71	15.40	3.02	9.22–21.58
T4	23.75	5.38	12.61–34.88	10.67	5.98	0.00–23.01
STAI-12						
T1	20.73	1.88	16.88–24.59	25.07	1.88	21.21–28.92
T2	19.57	2.09	15.27–23.87	20.34	2.05	16.10–24.57
T3	17.47	1.20	15.02–19.92	15.87	1.20	13.42–18.32
T4	17.76	1.60	14.39–21.12	18.68	1.74	15.05–22.30

Estimated marginal means for PTSS-10, IES-15, and STAI-12

**Fig. 1:**
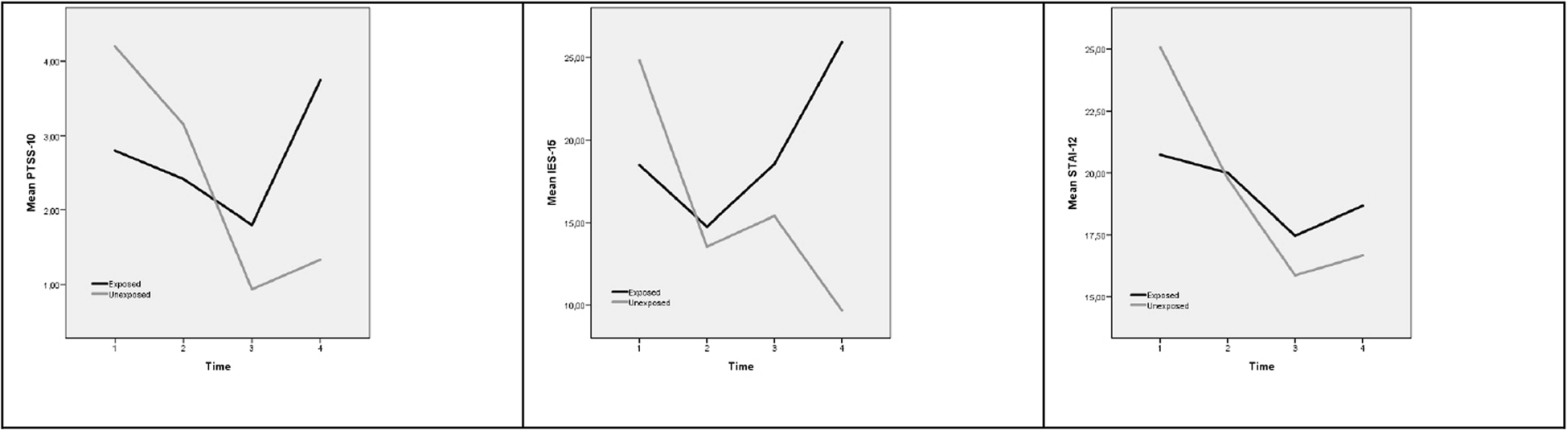
Changes in mean symptoms of posttraumatic stress, distress and anxiety symptoms by the PTSS-10, IES-15 and StAI-12. Time 1 (T1), Time 2 (T2), Time 3 (3) and Time 4 (T4). Values presented as estimated means with 95% CI from linear mixed models. High scores represent more symptoms of posttraumatic stress, distress and anxiety

As mentioned above, PTSS-10 did not reveal any statistically significant differences between the groups; there was, however, a significant effect of time. The *M*-levels of PTSS-10 declined over time, *p* = 0.001, for both groups, and the shape of the time trajectories showed a statistically significant difference between the groups (*p* = 0.013 for interaction term time*group) (Fig. 1).

The IES-15 did not reveal any differences between groups; however, there was a significant effect of time. The *M*-levels of IES-15 declined over time, *p* = 0.026, for both groups. The time trajectories tended to differ between groups; this did not, however, reach the level of statistical significance (Fig. 1).

Lastly, the STAI-12 did not reveal any differences between groups; however, there was a significant effect of time. The *M*-levels of STAI-12 declined over time, *p* = 0.001, for both groups. The shape of the time trajectories was not different between the groups (Fig. 1).

In 2016 (T4), 5/12 (42%) in the exposed group reported current PTS-symptoms (PTSS-10 ≥ 4), one half reported distress symptoms (IES-15 ≥ 26) and none reported anxiety symptoms (STAI-12 ≥ 30) above cut-off points, which would indicate need for psychological referral (Table 2). All (T1-T4) caseness numbers are displayed in Table 2.

Although not significant (all *p* > 0.05), the unexposed group reported lower proportions of individuals above cut-off points for almost all instruments, except for STAI-12, compared to the exposed group at T4.

Further, binary logistic regression analysis revealed no difference in odds to score above the cut-off between the groups for PTSS-10 (OR = 1.06, 95%CI [0.45–2.46], *p* = 0.901). The odds to score above the cut-off were lower for T2 and T4 compared to the T1 measurements; however, the difference did not reach the level of statistical significance. The odds to score above the cut-off were significantly lower at T3 compared to T1 (OR = 0.25, 95%CI [0.08–0.79], *p* = 0.018). The soldiers were about 75% less likely to score above the cut-off at T3 compared to T1 (Table 4).

**Table 4:** Binary logistic regression analysis

	PTSS-10			IES-15		
	OR	95% CI	*P*-values	OR	95% CI	*P*-values
Group						
Unexposed (ref.)	1					
Exposed	1.06	0.45–2.46	0.901	0.59	0.24–1.45	0.249
Time						
T1 (ref.)	1					
T2	0.39	0.13–1.20	0.101	0.10	0.02–0.49	< 0.01
T3	0.25	0.08–0.79	< 0.05	0.34	0.11–1.05	0.060
T4	0.40	0.12–1.32	0.132	0.54	0.17–1.76	0.309

Odds for scoring above the cut-off for PTSS-10 and IES-15. Binary logistic regression analysis

For the IES-15 there was no difference in odds to score above the cut-off between the groups (OR = 0.59, 95%CI [0.24–1.45], *p* = 0.249). The odds to score above the cut-off were lower for T2, T3 and T4 compared to the T1 measurements; however, the difference did not reach the level of statistical significance for T3 and T4. The odds to score above the cut-off were significantly lower at T2 compared to T1 (OR = 0.10, 95%CI [0.02–0.49], *p* = 0.005). The soldiers in both groups were about 90% less likely to score above the cut-off at T2 compared to T1 (Table 4). However, the odds were similar at T3 and T4 compared to T1 (all *p* > 0.05).

For the instrument STAI-12, there were too few individuals above the cut-off, therefore the model could not be fitted.

## Discussion

To our best knowledge, the present study was the first to investigate long-term mental health symptoms over three decades after an avalanche disaster. The study aimed to compare possible changes between exposed and unexposed soldiers experiencing an avalanche. The main finding was significant effect of time, where the adjusted mean levels for all measures declined over time for both groups. The time trajectories for PTSS-10 was significantly different between the groups, indicating an U-shaped course for the exposed group during the observed 30 years.

Several studies claim that individuals exposed to multiple PTE types may be at risk of more severe posttraumatic stress symptoms [6–11]. Our study shows no statistically significant differences between the groups regarding lifetime experienced PTEs. Our findings show that the exposed group reported almost the same proportion of PTEs in their lifetime as the unexposed group. These findings are not in accordance with those of Thordardottir and colleagues [20] and Benjet and colleagues [12], who argue that survivors experience more PTEs, and have more PTSD-symptoms, compared to unexposed individuals [12, 20]. Kessler, Sonnega, Bromet, and Hughes [56] claim that one explanation to a trend where survivors experience more PTEs, may be that previous PTEs are a risk factor for additional PTEs. However, the present study shows a large proportion of both the exposed (67%) and the unexposed (56%) soldiers having experienced one or more PTEs before or after the disaster (Table 1). These findings may indicate that the unexposed soldiers have the same pattern over time regarding PTEs. On the other hand, Bøe and colleagues [17] report findings contrary to Thordardottir and colleagues [20] and Benjet and colleagues [12] in their 27-year follow-up study. Bøe and colleagues [17] found additional traumatic exposure reported more frequently in the unexposed group. Bøe and colleagues [17] argue that this may be explained by survivors’ adaption to more secure lifestyles, thus reducing their risk of additional traumas. Another explanation might be experience bias making survivors report fewer traumatic experiences, as other PTEs may appear less important than the huge disaster experience [17]. Why our findings indicate almost the same proportion of PTEs in both groups is unclear. It may be a result of serving in the same platoon, being the same age and gender, undergoing the same selection procedures and, of course, both groups were closely related to the disaster, directly or indirectly. An important recent study by Kessler and colleagues [8] highlights that people exposed to earlier traumas are at significantly increased risk of subsequent traumas. This pattern of increased risk of trauma exposure was attributed to differences in individual lifestyles, life circumstances, coping resources and predispositions [8]. This might also be an explanation to the present study regarding the relative high proportion of self-reported PTEs in both groups. Lastly, it is noteworthy that, despite the proportion of PTEs in both groups being similar, the exposed group seem to have higher *M*-levels of PTSD-symptoms and proportion above cut-off (measured by PTSS-10 and IES-15), albeit not significant, compared to the unexposed group at T4.

A possible explanation for the non-significant differences in PTSD-symptoms (measured by PTSS-10 and IES-15) between the two groups in our study, may be related to the fact that the soldiers in the exposed and unexposed group served in the same platoon and that they knew each other very well. Therefore, the exposed and unexposed soldiers were affected with the trauma directly or indirectly. Thus the unexposed soldiers could be considered as victims (although indirectly). A previous study, May and Wisco [57] supports an assumption that level of direct and indirect exposure to trauma may affect individuals regardless of exposure impact.

The exposed group reported that the disaster had a significantly more pronounced negative impact on their physical and mental health compared to the unexposed group, which may be a consequence of the severity of the disaster. These findings are in line with previous studies that claim strong association to type and duration of exposure for the incidence and prevalence of psychopathology post-disasters [6–10]. It is here important to mention that 16 soldiers in the platoon died, and 14 of 15 soldiers in the exposed group were buried by the avalanche. Further, Rostrup, Gilbert, and Stalsberg [58] and Stalsberg and colleagues [59] reported a considerable proportion of physical injuries in the exposed group after the avalanche. The Piper Alpha study may support the findings that disasters may affect the mental health of survivors with physical injuries more negatively. The same study reported high rates of physical injury (83%) directly after the disaster, and high prevalence rates of PTSD (21%) 10 years post-disaster [60, 61].

Several findings in the present study regarding background characteristics are supported by Thordardottir, Hansdottir, Shipherd, and colleagues [43] in their 16-year follow-up study among avalanche survivors. Some previous military research on PTSD and other mental disorders in males also support similar findings. The military studies of Hougsnæs and colleagues [5], Iversen and colleagues [62] and Buckman and colleagues [63] report PTSD and other common mental disorders as more frequent in single males with lower education, age and rank.

PTSD-symptoms were present in many soldiers in both groups in the immediate aftermath. The severity and intensity of reactions seemed to affect the unexposed group more heavily at first (T1-T2) [40]. Herlofsen’s [40] interpreted this as due to an enforced passivity prohibiting the unexposed soldiers from joining the search party and working through their emotional state the first days following the disaster. Previous studies have reported similar findings [64, 65], but another study on unexposed soldiers following an avalanche reporting opposite findings [41]. However, other research on the negative impact of indirect exposure to trauma [57, 66] may be in accordance with Herlofsen’s findings [40].

Symptoms of PTS, distress and anxiety exhibited by the exposed and unexposed soldiers decreased during the first year after trauma (T1-T3), and there was a decrease, but not significant, for the unexposed soldiers from T1 to T3 regarding the posttraumatic stress (PTSS-10, IES-15) and anxiety (STAI-12) mean symptom scores. This may point to an ability to work through their emotional state during the first year after the accident and to not having a direct life-threatening experience.

The symptoms remained fairly stable thereafter for the unexposed group (T3-T4), but increased again at T4 for the exposed group. We did not observe a statistically significant difference in PTSS-10, IES-15 and STAI-12 *M*-scores beween the groups. However, our study may illustrate a tendency that the exposed soldiers have a higher PTSS-10, IES-15 and STAI-12 *M*-score, and a higher proportion of soldiers above cut-off points for the PTSS-10 and IES-15 than the unexposed soldiers, which indicate psychological referral 30 years post-disaster. On the other hand, our study showed that the effect of time was statistically significant in both groups regarding all measures, with *M*-levels of PTSS-10, IES-15 and STAI-12 declining over time. The shape of the time trajectories for PTSS-10 was also significantly different between groups, with course pattern of PTSS-10 symptoms increasing in the long-term for the exposed group. The IES-15 trajectories for the exposed group showed the same trend, but did not reach the level of statistical significance.

These results are mostly in line with previous short-term studies finding a marked decrease in PTSD-symptoms after traumatic events [15, 17, 20, 67–69]. Morina, Wicherts, Lobbrecht, and Priebe [70] claim that PTSD related to natural disasters has the highest mean of remission rates (60%) over time, compared to PTSD related to physical diseases (31.4%). It is thus noteworthy that the present study shows no decline after the first year (T3-T4) for the exposed group. However, no other avalanche study has followed up survivors over three decades. These findings are therefore of great importance for health authorities planning appropriate follow-up, and to prepare individuals for a possibly long-term journey after exposure.

There was an increase in PTSS-10, IES-15 and STAI-12 *M*-scores from T1 to T4 in the exposed group, which did not differ significantly from the unexposed group. These findings are contrary to many long-term studies on survivors [15–17, 20, 34, 60, 71–74]. Our findings may be supported by Kessler and colleagues [8], who argue that mean PTSD-symptoms duration is considerably longer than previously recognized, although a considerable minority of PTSD cases remits short time after onset. The present study’s findings may indicate that especially the exposed soldiers, carry a burden in the long-term perspective with negative PTSD-symptoms and anxiety symptoms 30 years post-disaster. This may be supported by previous studies claiming that PTSD-symptoms may occur soon after trauma or may be delayed (late-onset), sometimes for years [75]. However, many survivors will never experience, or be given an opportunity to report, all the symptoms for a full diagnosis of PTSD, but have subsyndromal or sub-threshold PTSD, which may impair functioning close to a fully diagnosed PTSD [76–79]. Further, Macleod [80] and Port, Engdahl, and Frazier [81] suggest that trauma-related psychopathology may follow a U-shaped course, a pattern supported in the present study.

Macleod [80] and Port and colleagues [81] report high levels of negative mental health symptoms immediately after trauma, declining during the years of work life but possibly returning as the survivors cope with age-related issues and transition into retirement. In the present study it is not known if such factors affect the level of negative mental health symptoms reported among the exposed 50-year old soldiers.

A significant difference between the groups was notable regarding the shape of the time trajectories for the PTSS-10, and the same trend was seen in the shape of the time trajectories for the IES-15, however not significant.

The present study indicates a higher proportion of exposed soldiers suffering from severe and intense PTSD-symptoms above cut-off points (PTSS-10 = 42%; IES-15 = 50%), compared to the unexposed soldiers (PTSS-10 = 11%; IES-15 = 11%). These findings, 30 year post-disaster, are exactly the same proportions above cut-off (PTSS-10, IES-15) as the exposed soldiers reported four days post-disaster (T1). This is not in accordance with what Bøe and colleagues [17] and Thordardottir and colleagues [20] report in their long-term follow-up studies. Bøe and colleagues [17] reported the incidence of PTSD (early onset) to be 22.9% after the disaster, and after 27 years the prevalence showed that just 6.3% of the male survivors had a full PTSD diagnosis. The same pattern was reported by Thordardottir and colleagues [20] in their 16 year long-term follow-up study. Further, Thordardottir and colleagues [20] emphasize that Asmundsson and Oddsson [38] and Finnsdottir and Elklit [39] reported that approximately 40% of the survivors suffering from PTSD the first 10 weeks to 14 months after the avalanches. These rates of PTSD declined in the long-term, with 12% of the male survivors suffering from avalanche-specific PTSD symptoms above the clinical cut-off 16 years post-trauma [20].

Thordardottir and colleagues [20] report higher levels of PTSD, while Bøe and colleagues [17] found lower levels than Lassemo and colleagues [7] estimated regarding risk for PTSD after a natural disaster for the general male population in Norway. Lassemo and colleagues [7] estimated that 9.1% of the male population would fill the diagnostic criteria of risk for PTSD after such disasters. These findings and estimates (6.3, 9.1 and 12%) may be lower than we can expect in our exposed male sample when there is no decline in the proportion above cut-off (PTSS-10 and IES-15) 30 years post-disaster, compared to data from T1.

However, it is important to emphasize that the present study uses just a few screening tools that may be efficient for identifying individuals at risk of psychopatologhy, and not structural clinical interviews or diagnostic tools for specific psychiatric diagnoses, like Bøe and colleagues [17]. This may give the present study a false high understanding of the proportion of soldiers with psychopathology when considering only current mental health symptoms above cut-off point, rather than investigating for specific psychiatric diagnoses with diagnostic tools and clinical interviews [82]. The picture may, however, be right, but the proportion of mental impairment among the soldiers both in the exposed and unexposed group may be even higher than expected if the non-responders had been included. Morina and colleagues [70] and Weisaeth [83] claim that the effect of non-participation may be an underestimation of severity and intensity of negative mental health symptoms.

Despite our findings indicating high level PTSD-symptoms among the exposed soldiers, none of them, and just one of the unexposed soldiers, score above the cut-off point regarding anxiety symptoms. These findings are not in accordance with some studies demonstrating the importance of general psychopathology, i.e., subsyndromal PTSD, depression, and anxiety disorders as the most prevalent conditions among survivors in the long-term perspective [17, 84]. The low proportion of anxiety symptoms above cut-off in our study may also be an expression of not using structural clinical interviews or diagnostic tools.

### Strengths and limitations

One strength of this study is the long-term follow-up of an avalanche disaster across three decades. Another is the use of standardized, validated measures and the mixed model (LMM) and binary regression analyses, enabling us to model longitudinal data.

This research was, however, conducted on a small sample, from an exclusive group of young Norwegian male soldiers, and the generalizability is likely limited to selected well-trained males; no female soldiers were exposed to this natural disaster. Several studies have revealed significant sex differences in response to traumatic events [85–87]. However, Thordardottir, Hansdottir, Shipherd, and colleagues [43] found no significant sex differences in the prevalence of PTSD 16 years after an avalanche.

Small sample sizes may evoke skepticism about whether the collected data can be subjected to a statistical test. Hackshaw [88] claims that the main problem with small studies is interpretation of results, in particular *p*-values and CIs. Any generalization of this study’s results to populations other than selected well-trained males should be done with care. The normality assumptions were tested by means of visual inspection of the residual plots. The model fit was good and the residuals followed normal distribution. The homogeneity of variance was also acceptable. According to our power calculations we would require 25 (PTSS-10), 23 (IES-15) and 121 (STAI-12) in both groups to reveal our findings as statistically significant with anticipated effect sizes as defined by Jacob Cohen [89], being medium (d = 0.5, PTSS-10) and small (d = 0.4, IES-15 and d = 0.4, STAI-12) [89]. Our analyses would require a higher sample size to reveal the main findings as statistically significant. However, due to ethical reasons it was important to present the results despite some of them being largely descriptive. Though the sample size is limited, it is important to emphasize that this study’s strengths are a homogeneous group and an almost complete 30 years follow-up.

The effect of non-participation may be an underestimation of severe and intense negative mental health symptoms. Previous studies claim that people experiencing PTSD-symptoms are less likely to answer follow-up studies [70, 83].

The current study is limited by lack of information on pre-disaster health status and the retrospective design. It is, however, important to emphasize that procedures for personnel selection and medical standards in the Norwegian Armed Forces make it fair to assume that no serious psychopathology was present pre-disaster. The retrospective design also introduces the possibility of recall bias when relating to one particular traumatic event. Another possible limitation is the 30 year span between the last two measure points from 1987 to 2016. This may reflect fluctuations this study is unable to detect.

Another possible study limitation is true symptom deviation, as the study relies on self-report rather than physical examinations and diagnostic tools [82]. An essential strength of this study is its indication of how PTS-symptoms, distress and anxiety symptoms may change over a very long time in a sample exposed directly or indirectly to a Criterion A-event [90]. We recommend long-term follow-up studies after life-threatening events in order to shed light on possible physical, mental and social impairment. In addition to standardized measures, qualitative studies may be valuable in this regard.

## Conclusion

This study did not reveal any significant differences in the PTSS-10, IES-15 or STAI-12 adjusted mean levels or scores above cut-off point between the exposed and unexposed groups. However, the study revealed a significant effect of time – the adjusted mean levels for all measures declined over time for both groups. Lastly, the shape of the time trajectories for PTSS-10 was significantly different between the groups, indicating an U-shaped course for the exposed group during the observed 30 years. For the IES-15, our data revealed a similar, but not statistically significant, trend.

This unique long-term study emphasizes that the course of PTS-symptoms (PTSS-10), distress (IES-15) and anxiety (STAI-12) symptoms may persist, and even increase, in selected and trained military personnel 30 years after exposure to a natural disaster. These findings may also be of great importance for health authorities planning appropriate follow-up, and to prepare individuals for a possibly long-term journey after exposure.

## Data Availability

The raw data is confidential and cannot readily be shared. Data may be shared with researchers obtaining permission from the Norwegian Regional Comittee for Medical Ethics and Norwegian Armed Forces Joint Medical Services, Institute of Military Psychiatry. After permission have been obtained, data can be made available from The Norwegian Armed Forces Joint Medical Services, Institute of Military Psychiatry, contact Lars-Petter Bakker: lpbakker@mil.no

## Abbrevations

CI: confidence intervals
IES-15: Impact of Event Scale-15
LMM: linear mixed model
M: Mean
OR: odds ratios
PTE: potentially traumatic event
PTS: Posttraumatic stress
PTSD: Posttraumatic stress disorder
PTSS-10: Posttraumatic Symptom Scale-10
SD: Standard deviation
STAI-12: State Anixiety/Agression Inventory
T1: Time 1
T2: Time 2
T3: Time 3
T4: Time 4
